# Is It Possible to Improve Motor Competence through a Structured Balance Bike Program in Preschool Children Aged 3 to 6 Years?

**DOI:** 10.3390/sports12030083

**Published:** 2024-03-15

**Authors:** Rubén Navarro-Patón, Fabián Canosa-Pasantes, Marcos Mecías-Calvo, Víctor Arufe-Giráldez

**Affiliations:** 1 Facultade de Formación do Profesorado, Universidade de Santiago de Compostela, 27001 Lugo, Spain; ruben.navarro.paton@usc.es; 2 Facultad de Ciencias de la Educación, Universidad de A Coruña, 15008 A Coruña, Spain; fabian.canosa@udc.es (F.C.-P.); v.arufe@udc.es (V.A.-G.)

**Keywords:** specific intervention program, preschool children, manual dexterity, aiming and catching, balance, Movement Assessment Battery for Children-2 (MABC-2)

## Abstract

This study aimed (1) to investigate the effect of a brief 9-week bike program on preschool motor competence levels and (2) to know the effects of the intervention program on gender. A total of 98 schoolchildren (3.86 ± 0.91 years) from La Coruña (Spain) participated. A pre–post-test quasi-experimental design was used with a control group (CG) made up of 39 students (18 boys) and an experimental group (EG) made up of 59 students (29 boys). Data were gathered using the Movement Assessment Battery for Children-2 (MABC-2). The data demonstrate that the CG and the EG differ significantly from one another before the intervention program in favour of CG in manual dexterity (MD; *p* < 0.001), balance (Bal; *p* = 0.003), total test score (TTS; *p* = 0.001), and total percentile score (TPS; *p* < 0.001), except in aiming and catching (A&C, *p* = 0.588). After the application of the 9-week intervention program based on the balance bike, these differences disappear (i.e., MD (*p* = 0.166), A&C (*p* = 0.372), Bal (*p* = 0.219), TTS (*p* = 0.306), and in TPS (*p* = 0.259)). The information gathered indicates that implementing intervention programs such as the one applied in this study could have a positive impact on improving motor competence in preschool-aged boys and girls, increasing its level.

## 1. Introduction

Motor competence (MC), understood as the ability to perform coordinated and effective movements [1], is a fundamental component in the holistic development of a human being and their quality of life [2]. It must be taken into account that many children’s learning is based on motor and gross motor skills [3], which can be classified as locomotive (i.e., running, jumping…), manipulative (i.e., throwing, receiving, etc.), and balance (i.e., static, dynamic) [4].

This MC can be acquired in a rudimentary way through the natural development and maturation of the person [5,6], but if optimal development is sought, continuous interaction with a physical and social environment that stimulates it is necessary [7]. In addition, adequate feedback and instruction is also needed [4] through learning environments with structured programs [8,9,10,11,12,13], and with a specific objective [4]. In this sense, it must be taken into account that the first 5 years of a child’s life are a sensitive period for the development of their MC, which must be acquired in structured learning environments (i.e., physical education classes (PE)) or recess or school breaks [9,13,14]. Despite this, the World Health Organization (WHO) indications for the practice of physical activity for preschool children (<5 years) focus on physical activity levels [15] and the components of the free play [16], with no specific recommendations for the development of MC at these ages.

Recent research indicates that the development of MC affects cognitive development [17], active participation in physical activities and sports [18], and overall quality of life and health (i.e., less overweight [19]), the delay of which could have negative effects [20]. Therefore, the acquisition of MC should be the highest-priority goal for the implementation of specific programs [21], because a mature form of MC is less likely to be achieved without adequate practice, stimulation, and feedback [4]. On the other hand, the field of MC has experienced important advances in terms of evaluation and intervention programs, which has allowed a more personalized and effective approach to improve it in children without disabilities and of both genders, both in the short term (4–8 weeks) [10,11,12] and in the long term (≥6 months) [22,23], although shorter-duration interventions (4 weeks to 5 months) showed better results than longer-duration interventions (>6 months) [23] in improving MC. For this reason, if we want to contribute to the development of MC [24,25], more research is needed in this regard, taking advantage of the fact that, owing to the substantial amount of time that children spend in schools, these organized programs are thought to be best implemented and developed there [26], even while some authors have attested to the presence of specific structural or material restrictions in educational institutions, which may be a reason to prefer one type of game over others, causing sliding skills such as skating or riding a skateboard, bike, or tricycle not to be promoted in school [27,28]. 

In this context, research on the possibilities offered by the balance bike in preschool children is very limited and scarce to date [29,30]. In the studies of Kavanagh et al. [29,30], significant correlations were found between the ability to ride a balance bike with the three components of basic motor skills (locomotion, object control, and balance). Despite this, cycling is considered a standalone fundamental movement skill [30], but this can be combined with locomotion, object control, and stability to produce an overall MC composite score. Therefore, due to limited scientific evidence and the fact that combining cycling with all three MC components can help increase fundamental movement skills and has been understudied, the purposes of this study were (1) to know the effect of a 9-week intervention program with balance bikes on MC levels in preschoolers aged 3 to 5 years; (2) to know the effects of the program intervention on gender. Therefore, it is suggested that toddlers who utilize balance bikes as part of the structured program will register a considerable improvement in their MC levels, independent of gender.

## 2. Materials and Methods

### 2.1. Study Design

This was a quasi-experimental study comparing 2 groups (Control vs. Experimental) with pre- and post-test measures based on MABC-2 variables and gender (boys vs. girls).

### 2.2. Participants

The group of participants in the incidental sample was made up of preschool children from 1 educational centre in Galicia (Spain). The inclusion criteria to participate in this study were (1) present informed consent signed by parents or legal guardians; (2) complete the entire process (initial test; 9-week period; final test); and (3) not suffer any illness or difficulty (physical or mental) that prevents participation in the MABC-2 tests. A total of 115 preschoolers between 3–5 years old were invited to participate, of which twelve were removed for not finishing the complete process and nine were removed for not having their legal guardians’ signed informed consent. Finally, the sample was made up of 98 preschoolers divided into two analysis groups, the CG (n = 39) and the EG (n = 59). The participants correspond to fourth (n = 48), fifth (n = 15), and sixth (n = 31) grade students of early childhood education. Regarding the gender variable, 48% of the participants were boys (n = 47) and 52% were girls (n = 51), distributing 18 boys and 21 girls in the CG and 29 boys and 30 girls in the EG.

### 2.3. Tools and Measurements

As an instrument for assessing MC, the MABC-2 adapted to the Spanish context by Graupera and Ruíz [31] was used, which has been shown to be feasible [31,32,33,34] and reliable [35] to identify changes in MC over time in preschool children. 

**Figure 1:**
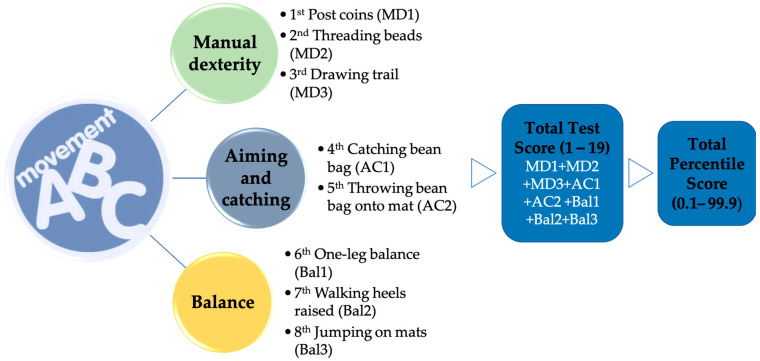
MABC-2 test dimensions (ranges).

Eight standardized examinations covering three distinct skills comprise this battery (Figure 1). Following the completion of all tests, the scale scores for each test were collected, and the scale and percentile scores of the total test score were obtained along with their corresponding scale and percentile scores.

### 2.4. Procedures

The goal was communicated to the school administration when they were contacted. The parents and/or legal guardians of the school-aged children were then given a study information sheet and asked to provide informed consent in order for them to participate. The data were gathered after they were approved. Following acceptance and acquisition of the parents’ or legal guardians’ signed informed consent, the required sociodemographic information (age and gender) was collected, and the preschoolers were assessed using the MABC-2 battery using standardized equipment and applied by trained evaluators. Each preschooler was evaluated individually, wearing comfortable clothing in a school room. Before performing each of the tests, the schoolchildren performed up to two attempts of each test where they were corrected by the evaluators. During the test (assessment), no instructions were administered. 

After the application of the MABC-2 battery, the students were assigned to the control (CG) or the experimental group (EG), according to the natural classroom to which they belonged, so that the balance bike intervention was easier to apply.

For the EG, the main researcher, a PE graduate with 15 years of experience as a PE specialist teacher in early childhood and primary education, taught all the sessions of the balance bikes program. The intervention replaced the motor skills classes and consisted of 3 sessions of 50 min per week, for 9 weeks (Table 1). Each session began by adjusting the bike to the anthropometric characteristics of the preschoolers and putting on a helmet and disposable cap at the time of warm-up or reception (5 min), then 6 tasks related to the contents of that session (Table 1; 40 min), and a moment of cooling down or farewell (5 min).

For the CG, the PE teacher of each school continued with the plans for the dates on which the balance bike project was developed without altering its programming. The aspects that were developed focused on the basic knowledge of the three areas of knowledge related to motor skills and PE of the physical education curriculum in early childhood education in Spain (i.e., the body and its progressive control; healthy lifestyle habits for self-care and care of the environment; body dialogue with the environment; creative exploration of objects, materials and spaces; language and body expression [36]). The duration, frequency of the procedure, and exact content followed by the CG were not recorded. The teacher who taught the motor skills sessions in the CG did not know the EG intervention contents and procedures. 

Lastly, the MABC-2 battery was administered again the day after the intervention ended, to both the CG and the EG.

### 2.5. Ethics

The entire research project was completed in compliance with the Declaration of Helsinki. The national EDUCA platform’s Ethics Committee received the research protocol and accepted it on 16 October 2021 with code number 15/2021.

### 2.6. Statistical Analysis

Quantitative data are expressed in measures of central tendency (mean; standard deviation) and qualitative data in frequencies. Initially, the data’s normal distribution was examined using the Kolmogorov–Smirnov test. Before the intervention program, to determine whether the control and experimental groups were equivalent on gender and age, on the one hand, a T-test for independent samples was performed for age, and on the other, a chi-square analysis was performed to compare the distribution of the participants by gender. Subsequently, a three-way ANOVA was performed with a group factor (2 levels), a time factor (2 levels), and a gender factor (2 levels), with repeated measures on the time factor, (i.e., time (pre-test vs. post-test)), to analyse the possible main effect of these factors and the interactions on the dependent variables studied (MD, A&C, Bal; TTS and TPS). IBM SPSS software version 25 (SPSS v.25, IBM Corporation, New York, NY, USA) was used for statistical analysis with a significance level set at *p* < 0.05.

## 3. Results

A total of 98 schoolchildren (3.86 ± 0.91 years) from La Coruña (Spain) participated. These preschoolers were assigned to a control group (CG) made up of 39 students (18 boys) and an experimental group (EG) made up of 59 students (29 boys).

Table 2 shows the data of the variables studied from the MABC-2 battery both before and after the 9-week intervention of the CG and the EG. The Kolmogorov–Smirnov test revealed that the data followed a normal distribution (*p* > 0.050). At the beginning of the study, the CG and EG participants were comparable in terms of gender (*p* = 0.771) and age (*p* = 0.078), being homogeneous groups based on these variables.

### 3.1. Manual Dexterity (MD)

The results indicate that there was a statistically significant interaction of the group factor and the time factor (F(1, 94) = 20.123, *p* < 0.001, η^2^ = 0.176, 95% CI 3.350, 8.671) before the intervention, with higher scores in the CG (M = 29,154, SD = 5.81) than in the EG (M = 23.119, SD = 7.92). These differences disappeared once the intervention program was applied (*p* = 0.166). No statistically significant interaction was found before and after the intervention in the CG (*p* = 0.070) or the EG (*p* = 0.055).

On the other hand, there was a significant interaction of the factors group (CG vs. EG) × gender (boy vs. girl) × time (preintervention vs. post-intervention) in the manual dexterity variable (F(1, 94) = 9.405, *p* = 0.003, η^2^ = 0.091, 95% CI 2.103, 9.825) between the boys of the CG (M = 28.72, SD = 1.52) and the boys of the EG (M = 22.75, SD = 1.20) before the intervention. The same occurred if the girls from both groups were compared before the intervention (F(1, 94) = 10.792, *p* = 0.001, η^2^ = 0.103, 95% CI 2.396, 9.718), with higher scores for the girls from the CG (M = 29.52, SD = 1.41) than those of the EG (M = 23.46, SD = 1.18). Once the 9-week intervention period had elapsed, these differences disappeared both between boys (*p* = 0.695) and girls (*p* = 0.109). A significant interaction of the factors group × gender × time was also found only in boys of the CG when comparing before (M = 28.72, SD = 5.25) and after (M = 24.72, SD = 7.24) the intervention (F(1, 94) = 4.968, *p* = 0.028, η^2^ = 0.050, (95% CI 0.425, 7.353)), producing a decrease in the score achieved. No statistically significant interaction was found in the rest of the comparisons.

### 3.2. Aiming and Catching (A&C)

The results indicate that there was no statistically significant interaction in the group and time factors either before (*p* = 0.588) or after the intervention. Likewise, there were no statistically significant interactions in the CG before and after the intervention (*p* = 0.445) or in the EG (*p* = 0.672).

Finally, the results obtained indicate that there was no significant interaction of the factors gender (boy vs. girl) × time (preintervention vs. post-intervention) × group (CG vs. EG) in A&C, or among boys of the CG and the EG before (*p* = 0.240) or after (*p* = 0.178). The same thing happened if the girls from both groups were compared. There were no differences between them either before (*p* = 0.637) or after (*p* = 0.887).

In the interaction of the factors group (CG vs. EG) × gender (boy vs. girl) × time (preintervention vs. post-intervention), a significant interaction was not found either (i.e., CG before (boys vs. girls, *p* = 0.352); CG after (boys vs. girls, *p* = 0.624); EG before (boys vs. girls, *p* = 0.465); EG after (boys vs. girls, *p* = 0.260)), nor was a statistically significant interaction found in the comparisons of boys from the CG (before vs. after, *p* = 0.771); girls from the CG (before vs. after, *p* = 0.414); boys from the EG (before vs. after, *p* = 0.978); girls from the EG (before vs. after, *p* = 0.565).

### 3.3. Balance (Bal)

The results indicate that there was a statistically significant interaction of the group factor and the time factor (F(1, 94) = 9.216, *p* = 0.003, η^2^ = 0.089, 95% CI 1.153, 5.514) before the intervention, with higher scores for the CG (M = 33.258, SD = 0.83) than the EG (M = 29.924, SD = 0.69). These differences disappeared once the intervention program was applied (*p* = 0.219). Regarding the interaction between group and time, a statistically significant interaction was found in the EG before (M = 29.924, SD = 0.69) vs. after (M = 33.0.29, SD = 0.55) the intervention (F(1, 94) = 17.825, *p* < 0.001, η^2^ = 0.159, 95% CI −4.565, −1.645). No statistically significant interaction was found before and after the intervention in the CG (*p* = 0.347).

The results obtained indicate that there was a significant interaction of the factors gender (boy vs. girl) × time (preintervention vs. post-intervention) × group (CG vs. EG) in the Bal between the boys of the CG and the EG before the intervention (F(1, 94) = 5.774, *p* = 0.018, η^2^ = 0.159, 95% CI 0.665, 6.994), being higher in the boys of the CG (M = 32.278, SD = 1.25) than in boys of the EG (M = 29.44, SD = 0.06). These significant differences disappeared after the intervention (*p* = 0.685). No differences were found if girls from both groups were compared; there were no differences between them either before (*p* = 0.603) or after (*p* = 0.175).

In the comparisons of the interactions of the factors group (CG vs. EG) × gender (boy vs. girl) × time (preintervention vs. post-intervention), no differences were found within the children in the control group, whether the boys were compared with the girls before the intervention (*p* = 0.981) and after (*p* = 0.223). The same occurred in the comparison within the control group both before the intervention (*p* = 0.493) and after (*p* = 0.625).

Finally, a significant interaction of the factors group × gender × time was also found only in boys of the EG when they were compared before (M = 29.44, SD = 0.98) and after (M = 32.75, SD = 0.78) the intervention (F(1, 94) = 9.960, *p* = 0.002, η^2^ = 0.096, 95% CI −5.393, −1.228), producing a decrease in the score achieved. These differences also existed between girls in the EG before (M = 30.40, SD = 0.97) and after (M = 33.30, SD = 0.77) the intervention (F(1, 94) = 7.908, *p* = 0.006, η^2^ = 0.078, 95% CI: −4.948, −0.852).

### 3.4. Total Test Score (TTS)

The results indicate that there was a statistically significant interaction between the group factor and time factor (F(1, 94) = 12.896, *p* = 0.001, η^2^ = 0.121, 95% CI 4.020, 13.963) before the intervention, with higher scores for the CG (M = 79.96, SD = 1.94) than the EG (M = 70.97, SD = 1.57). These differences disappeared once the intervention program was applied (*p* = 0.306). Significant differences were found before and after the intervention in the EG (F(1, 94) = 9.247, *p* = 0.003, η^2^ = 0.090, 95% CI −8.449, −1.774), with lower scores before (M = 70.97, SD =1.57) than after (M = 76.08, SD = 1.51). No statistically significant interaction was found before and after the intervention in the CG (*p* = 0.501).

The results obtained indicate that there was a significant interaction of the factors gender (boy vs. girl) × time (preintervention vs. post-intervention) × group (CG vs. EG) in the TTS between the boys of the CG and the EG before the intervention (F(1, 94) = 8.277, *p* = 0.005, η^2^ = 0.081, 95% CI 3.239, 17.669), being higher in boys of the CG (M = 79.83, SD = 2.85) than boys of the EG (M = 69.37, SD = 2.42). These significant differences disappeared after the intervention (*p* = 0.604). These differences were also found among girls (F(1, 94) = 4.775, *p* = 0.031, η^2^ = 0.048, 95% CI 0.688, 14.370) before the intervention, being higher in the girls of the CG (M = 80.09, SD = 2.54) than in the girls of the EG (M = 72.56, SD = 2.21). These significant differences disappeared after the intervention (*p* = 0.346).

The results obtained indicate that there was no significant interaction of the factors group (CG vs. EG) × gender (boy vs. girl) × time (preintervention vs. post-intervention) in the TTS (i.e., GC before (boys vs. girls, *p* = 0.946); CG after (boys vs. girls, *p* = 0.156); GE before (boys vs. girls, *p* = 0.315); GE after (boys vs. girls, *p* = 0.187)). A statistically significant interaction was also not found in the comparisons of boys of the CG (before vs. after, *p* = 0.771); CG girls (before vs. after, *p* = 0.414); EG boys (before vs. after, *p* = 0.978); EG girls (before vs. after, *p* = 0.565).

Finally, a significant interaction of the factors group × gender × time was found only in the girls of the EG when comparing before (M = 71.56, SD = 2.11) and after (M = 78.10, SD = 2.12) the intervention (F(1, 94) = 5.512, *p* = 0.021, η^2^ = 0.055, (95% CI −10.213, −0.854)), producing an increase in the score achieved. In the rest of the comparisons, no statistically significant interaction was found.

### 3.5. Total Percentile Score (TPS)

The results indicate that there was a statistically significant interaction of the group factor and the time factor (F(1, 94) = 15.557, *p* < 0.001, η^2^ = 0.142, 95% CI 10.714, 32.435) before the intervention, having higher scores for the CG (M = 53.853, SD = 4.24) than the EG (M = 32.01, SD = 3.77). These differences disappeared once the intervention program was applied (*p* = 0.259). Significant differences were found before and after the intervention in the EG (F(1, 94) = 7.639, *p* = 0.007, η^2^ = 0.081, 95% CI −18.444, −3.023), with lower scores before (M = 32.27, SD = 3.44) than after (M = 43.01, SD = 3.77). No statistically significant interaction was found before and after the intervention in the CG (*p* = 0.400).

The results obtained indicate that there was a significant interaction of the factors gender (boy vs. girl) × time (preintervention vs. post-intervention) × group (CG vs. EG) in the TPS between the boys of the CG and the EG before the intervention (F(1, 94) = 7.706, *p* = 0.007, η^2^ = 0.076, 95% CI 6.275, 37.800), being higher in boys of the CG (M = 53.44, SD = 6.23) than boys of the EG (M = 31.40, SD = 4.91). These significant differences disappeared after the intervention (*p* = 0.460). These differences were also found among girls (F(1, 94) = 7.866, *p* = 0.006, η^2^ = 0.077, 95% CI 6.166, 36.058) before the intervention, being greater in girls of the CG (M = 54.26, SD = 5.77) than in girls of the EG (M = 33.15, SD = 4.80). These significant differences disappeared after the intervention (*p* = 0.389).

The results obtained indicate that there was no significant interaction of the factors group (CG vs. EG) × time (preintervention vs. post-intervention) × gender (boy vs. girl) in the TTS (i.e., CG before (boys vs. girls, *p* = 0.924); CG after (boys vs. girls, *p* = 0.254); EG before (boys vs. girls, *p* = 0.801); EG after (boys vs. girls, *p* = 0.189)). A statistically significant interaction was also not found in the comparisons of boys of the CG (before vs. after, *p* = 0.205); girls of the CG (before versus after, *p* = 0.893); boys of the EG (before vs. after, *p* = 0.236). Finally, a significant interaction of the factors group × gender × time was found only in the girls of the EG when comparing before (M = 33.15, SD = 4.83) and after (M = 48.01, SD = 5.28) the intervention (F(1, 94) = 7.444, *p* = 0.008, η^2^ = 0.079, (95% CI −25.669, −4.045)), producing an increase in the score achieved.

## 4. Discussion

To the best of the authors’ knowledge, the proposed study is the first to investigate whether the effects of a 9-week intervention program centred around balancing bikes on MC levels in Galician preschool children vary according to gender. The results obtained in the study indicate that a short-term program, based on balance bikes, with a frequency of 3 weekly sessions of 50 min improves MC in preschoolers with a percentile appropriate for their age [10,11], and in preschoolers below the 5th percentile (Developmental Coordination Disorder) [12], to the point of equalling the initial differences of those with a higher MC. For improving MC, it is necessary to develop specific structured intervention programs [11,12,21,37]. If the program, in addition to being structured, is implemented by a PE specialist, the children who receive it are more likely to improve their MC [11,22,23,37] than those who receive general PE classes taught by an early childhood education teacher [11].

Before the intervention, the CG had a higher MC in four of the five skills studied at a global level (i.e., manual dexterity; balance; total test score and total percentile score). According to gender, there were no significant differences between boys and girls, either overall or in either group (i.e., CG and EG), although girls were slightly superior in manual dexterity and balance, and in the total score of the test and in the total percentile score, both globally and in each of the groups (CG and EG), as in previous studies [10,11,38,39,40]. Aiming and catching scores tend to be higher in boys since they tend to show higher scores in control and manipulation skills [10,11,34,39,41,42,43]. However, the initial results of this research indicated that the scores in this skill were similar between boys and girls. 

Following the conclusion of the CG’s intervention period, the participants’ scores in several skills increased (i.e., aiming and catching; balance) and others even decreased (i.e., manual dexterity; total test score and total percentile score), although not significantly. This increase in the different MC test scores may be due in part to the growth and/or maturation of preschoolers [44] and not to the intervention in PE classes, since, as the children mature, their MC can improve without practice, but only little [22]. This is because for an improvement in MC to occur, scientific evidence indicates that structured and specific intervention programs are necessary [11,12,21,37]. According to gender, after the intervention, the similarities remain without differences in the CG and in the EG, although they improve compared to the initial assessment; these results are similar to those reported in the studies by Bolger et al. [45] and Cohen et al. [46]. 

The scores of the various dimensions in the EG significantly increased after the specified program was implemented through the use of balance bikes, indicating that the intervention program produced improvements in MC [10,11,12,29,30,42,47]. In this sense, we could assume that this improvement in MC scores could have occurred due to the growth and maturation of preschoolers [44], but if so, these significant improvements should have occurred in both the CG and in the EG. Although this intervention only lasted nine weeks, shorter than other interventions [23,37,48], significant improvements occurred in the EG. These improvements, in such a short time, could be related to better performance being achieved in the early stages of the intervention due to the ceiling effect [11,12,24], since a longer intervention time does not translate into better performance [22]. According to the results obtained, shorter interventions like ours could improve the MC of preschool children [11].

When performing the pre–post-test analysis, according to gender in the EG, the scores continued to be maintained with the same differences as before the intervention. Both genders obtained similar scores, although girls obtained slightly higher scores in all skills and overall scores, but without statistically significant differences, which may be because the ability to ride a bike can be considered an independent basic motor skill [30], although it produces improvements in balance and motor skills (object control, jumping, and running) [29]. Furthermore, these contents are poorly developed by early childhood education teachers in physical education sessions [49].

When the post-test comparison is made between the CG and the EG, the previous statistically significant differences disappear [21]. This increase in the EG scores occurs, above all, in manual dexterity and balance skills, and, consequently, the scores of the total test score and the total percentile score also increase significantly, as occurs in other studies [10,11,24,29,47,50,51,52].

This research has certain limitations, such as the limited sample size and its selection based on convenience, geographical proximity, and possibility of access. Furthermore, there is also no tool to specifically evaluate the quality of MC programs, so it is unknown if this intervention would have the same effect in other locations at a national and international level. On the other hand, a long-term follow-up of these schoolchildren has not been carried out to verify whether this intervention program, using balance bikes, maintains its long-term effect. Finally, it should also be noted that the multiple personal and environmental factors related to MC that can affect performance at a certain point in development were not taken into account [53].

As future lines of investigation, we propose the following: (1) carry out studies that use balance bikes as intervention programs in which medium–long-term follow-ups are carried out; (2) research studying the feasibility and possible improvements in CM in preschoolers who have CDC; (3) studies that include other personal factors (differences in maturity and motor experience, age, or gender) or environmental factors (possibility of practice or lack of physical activity) related to CM.

## 5. Conclusions

The results obtained support the initial hypothesis of this study, which stated that participating in a structured program using balance bikes would significantly improve the MC levels of the preschoolers who participated in it, regardless of gender. Even with these advancements, more research similar to the one suggested is required to determine whether these gains can be sustained over time. According to the study’s findings, preschoolers who use balance bikes as part of a specialized MC intervention can greatly increase their manual dexterity, aim and catch skills, balance, total test scores, and total percentile scores on the MABC-2 postintervention assessments. Therefore, it can be concluded that a balance bike program is viable and improves manual dexterity and balance. For this reason, the development of motor competence (MC) in children is one of the objectives of school PE, considering the development of this MC as a fundamental element within quality PE. This quality PE should provide an inclusive environment and qualified and meaningful opportunity for all children, so programs such as the one carried out in this research can contribute to this.

## Figures and Tables

**Table 1:** Contents to work on in the design of the project intervention sessions.

Week	Contents/Sessions	
1st	Information and familiarization	Session 1: Bike mascot, bike parts, and basic elements for greater safety.
		Session 2: Familiarization with the bike. Walking with the bike, getting on and off the bike. Carrying the bike from one side to another (swaying).
		Session 3: Walking with the bike, getting on and off the bike. Carrying the bike from one side to another (swaying).
2nd	Control of the exercise bike and first displacements	Session 4: Get on the bike and move with short steps. Getting on and off the bike.
		Session 5: Get on the bike and move with short steps. Getting on and off the bike.
		Session 6: Get on the bike and move with short steps. Getting on and off the bike.
3rd	Displacements in different directions	Session 7: Travel by bike from one place to another. Change of direction, turns, dribbling.
		Session 8: Travel by bike from one place to another. Change of direction, turns, dribbling.
		Session 9: Travel by bike from one place to another. Change of direction, turns, dribbling.
4th	Accelerate and brake	Session 10: Making changes from stopped to started. Acceleration on a straight line. Brake when faced with obstacles.
		Session 11: Making changes from stopped to started. Acceleration on a straight line. Brake when faced with obstacles.
		Session 12: Making changes from stopped to started. Acceleration on a straight line. Brake when faced with obstacles.
5th	Displacements with obstacles	Session 13: Movement avoiding obstacles (cones, cardboard boxes, etc.). Movement through different obstacles, narrow corridors, ramps, descents.
		Session 14: Movement avoiding obstacles (cones, cardboard boxes, etc.). Movement through different obstacles, narrow corridors, ramps, descents.
		Session 15: Movement avoiding obstacles (cones, cardboard boxes, etc.). Movement through different obstacles, narrow corridors, ramps, descents.
6th	Individual, competitive, cooperative and opposition games	Session 16: Games of personal improvement, cooperation, and opposition.
		Session 17: Games of personal improvement, cooperation, and opposition.
		Session 18: Games of personal improvement, cooperation, and opposition.
7th	Games and road education	Session 19: Games that involve the incorporation of signs and other aspects of road safety education.
		Session 20: Games that involve the incorporation of signs and other aspects of road safety education.
		Session 21: Games that involve the incorporation of signs and other aspects of road safety education.
8th	Games and road education	Session 22: Games that involve the incorporation of signs and other aspects of road safety education.
		Session 23: Games that involve the incorporation of signs and other aspects of road safety education.
		Session 24: Games that involve the incorporation of signs and other aspects of road safety education.
9th	Games and road education	Session 25: Games that involve the incorporation of signs and other aspects of road safety education.
		Session 26: Games that involve the incorporation of signs and other aspects of road safety education.
		Session 27: Games that involve the incorporation of signs and other aspects of road safety education.

**Table 2:** MABC-2 pre and post intervention of preschool children.

Variable		CG Pre (n = 39)	EG Pre (n = 59)	CG Post (n = 39)	EG Post (n = 59)
**Total score of manual dexterity**	boy	28.72 ± 5.25	22.75 ± 8.49	24.83 ± 7.94	24.03 ± 6.83
	girl	29.52 ± 6.35	23.46 ± 4.73	29.04 ± 5.57	25.93 ± 6.71
	all	29.15 ± 5.81	23.11 ± 6.79	27.10 ± 7.00	25.00 ± 6.78
**Total score of aiming and catching**	boy	19.11 ± 5.38	17.17 ± 5.25	17.69 ± 5.22	17.20 ± 6.84
	girl	17.47 ± 4.27	18.20 ± 6.13	18.06 ± 5.27	18.90 ± 5.51
	all	18.21 ± 4.80	17.69 ± 5.69	19.07 ± 4.87	18.06 ± 6.20
**Total score of balance**	boy	33.27 ± 4.68	29.44 ± 6.56	33.27 ± 4.26	32.75 ± 4.41
	girl	33.23 ± 5.56	30.40 ± 3.94	34.95 ± 4.59	33.30 ± 3.78
	all	33.25 ± 5.11	29.93 ± 5.36	34.17 ± 4.47	33.03 ± 4.08
**Total test score**	boy	79.83 ± 11.57	69.37 ± 14.57	75.88 ± 13.71	74.06 ± 12.75
	girl	80.09 ± 10.94	72.56 ± 10.44	81.23 ± 9.32	78.10 ± 10.62
	all	79.97 ± 11.09	71.00 ± 12.63	78.76 ± 11.71	76.11 ± 11.79
**Total percentile score**	boy	53.44 ± 30.30	31.40 ± 25.40	44.47 ± 30.83	38.01 ± 30.58
	girl	54.26 ± 27.04	33.15 ± 24.55	55.14 ± 27.65	48.00 ± 27.05
	all	53.88 ± 28.21	32.09 ± 24.77	50.21 ± 29.27	43.09 ± 29.03

Note: Data are presented as mean ± standard deviation mean.

## Data Availability

The data presented in this study are not available in accordance with Regulation (EU) of the European Parliament and of the Council 2016/679 of 27 April 2016 regarding the protection of natural persons with regard to the processing of personal data and the free circulation of these data (RGPD).
